# Experimental demonstration of spectral domain computational ghost imaging

**DOI:** 10.1038/s41598-021-87355-z

**Published:** 2021-04-16

**Authors:** Piotr Ryczkowski, Caroline G. Amiot, John M. Dudley, Goëry Genty

**Affiliations:** 1 Laboratory of Photonics, Tampere University, 33101 Tampere, Finland; 2 Institut FEMTO-ST, Université Bourgogne Franche-Comté CNRS UMR 6174, 25000 Besançon, France

**Keywords:** Imaging and sensing, Optical spectroscopy

## Abstract

We demonstrate computational spectral-domain ghost imaging by encoding complementary Fourier patterns directly onto the spectrum of a superluminescent laser diode using a programmable spectral filter. Spectral encoding before the object enables uniform spectral illumination across the beam profile, removing the need for light collection optics and yielding increased signal-to-noise ratio. In addition, the use of complementary Fourier patterns allows reduction of deleterious of parasitic light effects. As a proof-of-concept, we measure the wavelength-dependent transmission of a Michelson interferometer and a wavelength-division multiplexer. Our results open new perspectives for remote broadband spectral measurements.

## Introduction

Ghost imaging is an indirect measurement technique that uses the correlation between the intensity profile of a spatially-resolved light beam and the spatially-integrated intensity of the same beam transmitted through (or reflected from) an object to reconstruct an image of that object^1,2^. When first developed, the illumination patterns were based on random noise, which requires the beam intensity profile to be measured in a separate reference arm^3^. The image is then obtained from the correlation of the measured reference beam profiles and signals measured by a single pixel (integrating) detector placed after the object. Extensively studied to reconstruct spatial images, the technique has recently been extended to other domains including temporal^4–6^ and spectral domains^7–9^.

The need for distinct reference measurements of the illuminating patterns can be eliminated using a computational approach commonly referred to as computational ghost imaging or single-pixel imaging^3^. In this case, a set of specially designed (and stored) intensity masks are used for illumination, and it is then only necessary to use just one single-pixel detector to measure the integrated intensity after light interaction with the object. The image is obtained by solving a simple inversion problem. Using an appropriate set of (mathematically) orthogonal illuminating patterns, the computational imaging approach is significantly faster and yields enhanced signal-to-noise ratio as compared to random illumination ghost imaging. If the measured object is sparse, one can use compressed sensing techniques to reduce even further the number of distinct measurements^10,11^. Computational ghost imaging has been demonstrated both in the spatial and temporal domains^3,6,12^.

Computational imaging analysis has also been proposed in the wavelength domain using a liquid crystal cell to apply spectral masks^13,14^ in the detection plane to reconstruct its spectral response^15^. Subsequent studies have expanded the concept using compressed sensing approaches^16,17^, adding polarization analysis^17,18^ or to 2D measurements^19^. Here, inspired by Fourier transform interferometry, we present a new approach to sub-nm resolution computational ghost imaging in the spectral domain by modulating the spectrum of a broadband light source with harmonic series of sine and cosine patterns^11,20,21^. With this approach, one obtains directly the Fourier Transform coefficients of the object spectral response which can then is then retrieved by simple inversion, improving significantly the measurement speed as compared to when using random spectral fluctuations necessitating the use of a reference arm and subsequent correlation^7,8^. Applying complementary Fourier modulation patterns directly onto the light source spectrum has multiple benefits including uniform spectral illumination across the beam profile and reduction of stray light. The technique is especially suitable for remote sensing when spectrally-resolved measurements are not possible e.g. in the presence of strong scattering or low signal levels, and it also can be extended to 2D hyperspectral measurements^22,23^.

## Methods

We begin by describing the general principle of spectral-domain ghost imaging. Consider an object with a spectral response (transmission or reflection) $T(\omega _0+\Omega )$, where $\Omega$ is a relative optical frequency, spanning $\Delta \omega$ around a central frequency $\omega _0$. This spectral response can be decomposed onto a basis of (truncated) harmonic series of $N+1$ sine and cosine functions such that:

1 $$\begin{aligned} T(\omega _0+\Omega )= \sum _{n=0}^{N} a_{n}\cos \left( {\frac{2\pi n \Omega }{\Delta \omega }}\right) + \sum _{n=0}^{N} b_{n}\sin \left( {\frac{2\pi n \Omega }{\Delta \omega }}\right) , \end{aligned}$$

where $a_{n}$ and $b_{n}$ represent the *n* *th* cosine and sine Fourier coefficients, respectively, defined as

2 $$\begin{aligned} a_{n}&= \frac{2}{\Delta \omega }\int _{-\frac{\Delta \omega }{2}}^{+\frac{\Delta \omega }{2}}T(\omega _0+\Omega )\cos \left( {\frac{2\pi n \Omega }{\Delta \omega }}\right) d\Omega , \end{aligned}$$

3 $$\begin{aligned} b_{n}&= \frac{2}{\Delta \omega }\int _{-\frac{\Delta \omega }{2}}^{+\frac{\Delta \omega }{2}}T(\omega _0+\Omega )\sin \left( {\frac{2\pi n \Omega }{\Delta \omega }}\right) d\Omega . \end{aligned}$$

By illuminating the object with sinusoidal intensity patterns of different angular frequencies and measuring with a single-pixel detector the (spectrally) integrated intensity after transmission (or reflection) through the object, one can obtain the Fourier coefficients and thereby retrieve the object’s spectral response from Eq. (1). In practice, the modulation is encoded onto the spectral intensity of the light source, which means that the sinusoidal modulation (sine or cosine) has a DC component equal to source’s mean spectral intensity $I_{0}$. In order to eliminate the DC component, the object can be illuminated with complementary modulation patterns with reversed phase^12,24^:

4 $$\begin{aligned} I_n^{\pm c}(\omega _0+\Omega )&=I_{0}\left[ 1 \pm \cos \left( {\frac{2\pi n \Omega }{\Delta \omega }}\right) \right] , \end{aligned}$$

5 $$\begin{aligned} I_n^{\pm s}(\omega _0+\Omega )&=I_{0}\left[ 1 \pm \sin \left( {\frac{2\pi n \Omega }{\Delta \omega }}\right) \right] , \end{aligned}$$

and the spectral response is then retrieved from:

6 $$\begin{aligned} \begin{aligned} T(\omega _0+\Omega )&= \frac{1}{2I_{0}} \sum _{n=0}^{N} \left( a_{n}^{+}-a_{n}^{-}\right) \cos \left( {\frac{2\pi n \Omega }{\Delta \omega }}\right) \\&\quad +\frac{1}{2I_{0}} \sum _{n=0}^{N} \left( b_{n}^{+}-b_{n}^{-}\right) sin\left( {\frac{2\pi n \Omega }{\Delta \omega }}\right) , \end{aligned} \end{aligned}$$

where

7 $$\begin{aligned} a_{n}^{\pm }&= \frac{2}{\Delta \omega }\int _{-\frac{\Delta \omega }{2}}^{+\frac{\Delta \omega }{2}}T(\omega _0+\Omega )I_n^{\pm c}(\omega _0+\Omega ) d\Omega , \end{aligned}$$

8 $$\begin{aligned} b_{n}^{\pm }&= \frac{2}{\Delta \omega }\int _{-\frac{\Delta \omega }{2}}^{+\frac{\Delta \omega }{2}}T(\omega _0+\Omega )I_n^{\pm s}(\omega _0+\Omega ) d\Omega . \end{aligned}$$

Note that the use of complementary Fourier patterns further removes potential background parasitic light^12^. A total of 4N+2 patterns are then required to reconstruct the spectral transmission of the object (sine patterns for $n=0$ can be omitted), and the fact that one uses a truncated series limits the measurement spectral resolution to $\Delta \omega /\mathrm N$. Note that this constitutes a lower limit for the resolution and in practice the resolution can be further degraded due to the limited bandwidth of the programmable filter resulting in a loss of contrast of the probing patterns contrast at higher modulation frequencies.

**Figure 1:**
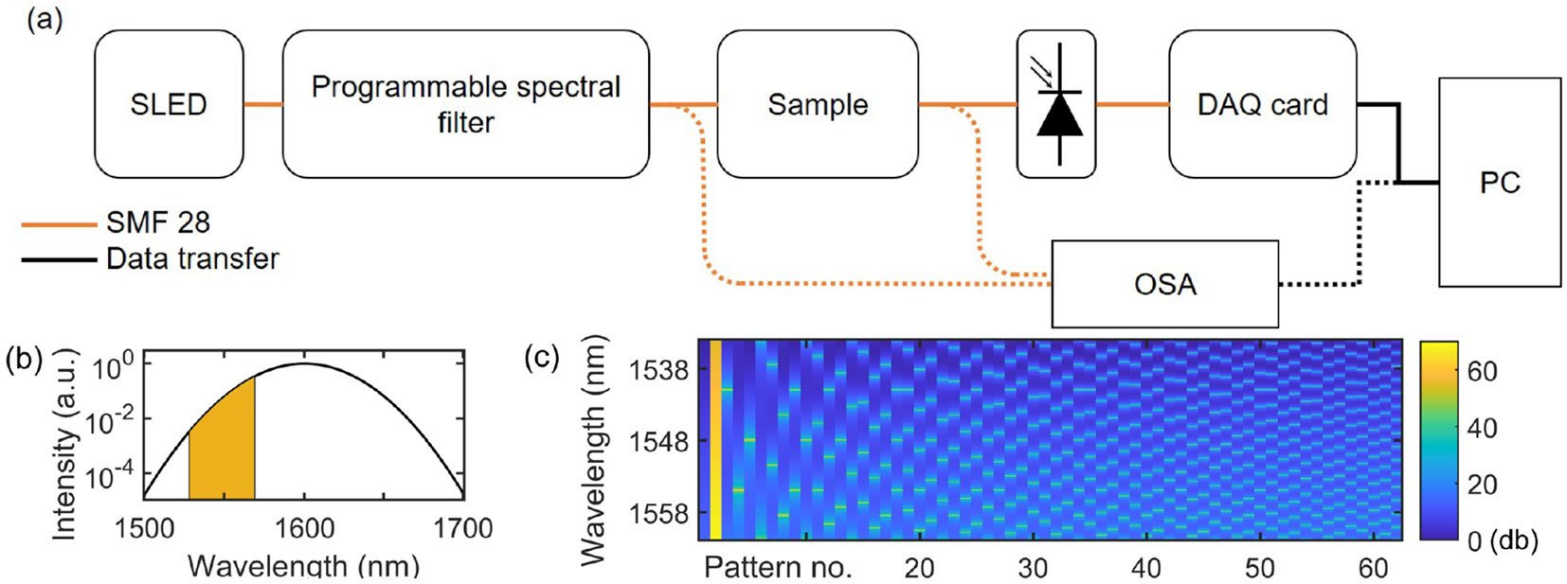
(**a**) Experimental setup. (**b**) SLED full spectrum with the wavelength band of the programmable spectral filter highlighted in orange. (**c**) Programmed spectral filter attenuation to generate the complementary Fourier pairs, here limited to 33 pairs patterns (N = 16) for clarity. The patterns are plotted in the following order: constant intensity ($I_0^{+c}$), zero intensity ($I_0^{-c}$), followed by complementary pairs of sine and cosine with increasing modulation frequency.

Our experimental setup is shown in Fig. 1a. Light from a fiber-coupled superluminescent diode (SLED) (Exalos ESL1620-2111) is directed through a programmable spectral filter (Finisar Waveshaper 4000s) which sequentially modulates the spectral intensity according to the complementary patterns described above (Fig. 1c). The filter uses a diffraction grating imaging setup coupled with a high-resolution liquid crystal on silicon optical processor to produce arbitrary spectral transfer functions (amplitude and phase) with bandwidth programmable in 1 GHz increment from 10 GHz to 1 THz over the 1527.5–1567.5 nm range. The output of the filter is collimated (or fiber-coupled) to illuminate the sample under test. Light after the sample is collected with a single-pixel large area detector (Thorlabs PDA50B-EC) with no spectral resolution. The wavelength-dependence of the source is pre-compensated by pre-normalizing the probing sinusoidal patterns to the SLED unmodulated spectrum (Fig. 1b). The electronic signal corresponding to each sinusoidal pattern is digitized with a DAQ card (NI USB-6212) and stored in a computer. The sequential feeding of modulated spectral patterns and data acquisition is controlled with a LabVIEW program. The spectral response is reconstructed by post-processing using Eq. (6). In order to validate the method, we also perform an independent reference measurement of the sample spectral response using an optical spectrum analyser (OSA, Ando AQ-6315B).

## Results

**Figure 2:**
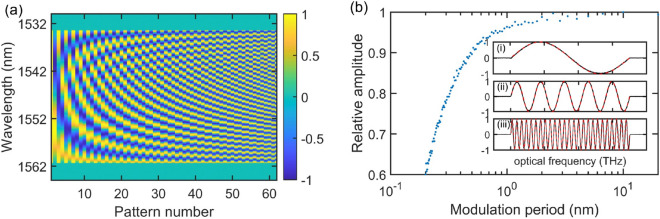
(**a**) First 61 sine and cosine complementary patterns measured individually with the OSA. The first pattern represents the unmodulated SLED spectrum (constant pattern) followed by pairs of sine and cosine patterns of increasing modulation frequency (decreasing modulation period). (**b**) Modulation amplitude of the 100 sine patterns as a function of modulation period, normalized to the highest amplitude value of the modulated pattern with lowest modulation frequency. The inset shows the measured sine patterns for three specific modulation frequencies 3.5 THz ($\sim$ 28 nm), 0.7 THz ($\sim$ 5.6 nm), and 0.14 THz ($\sim$ 1.1 nm) as solid black lines together with ideal sine functions as dashed red lines.

**Figure 3:**
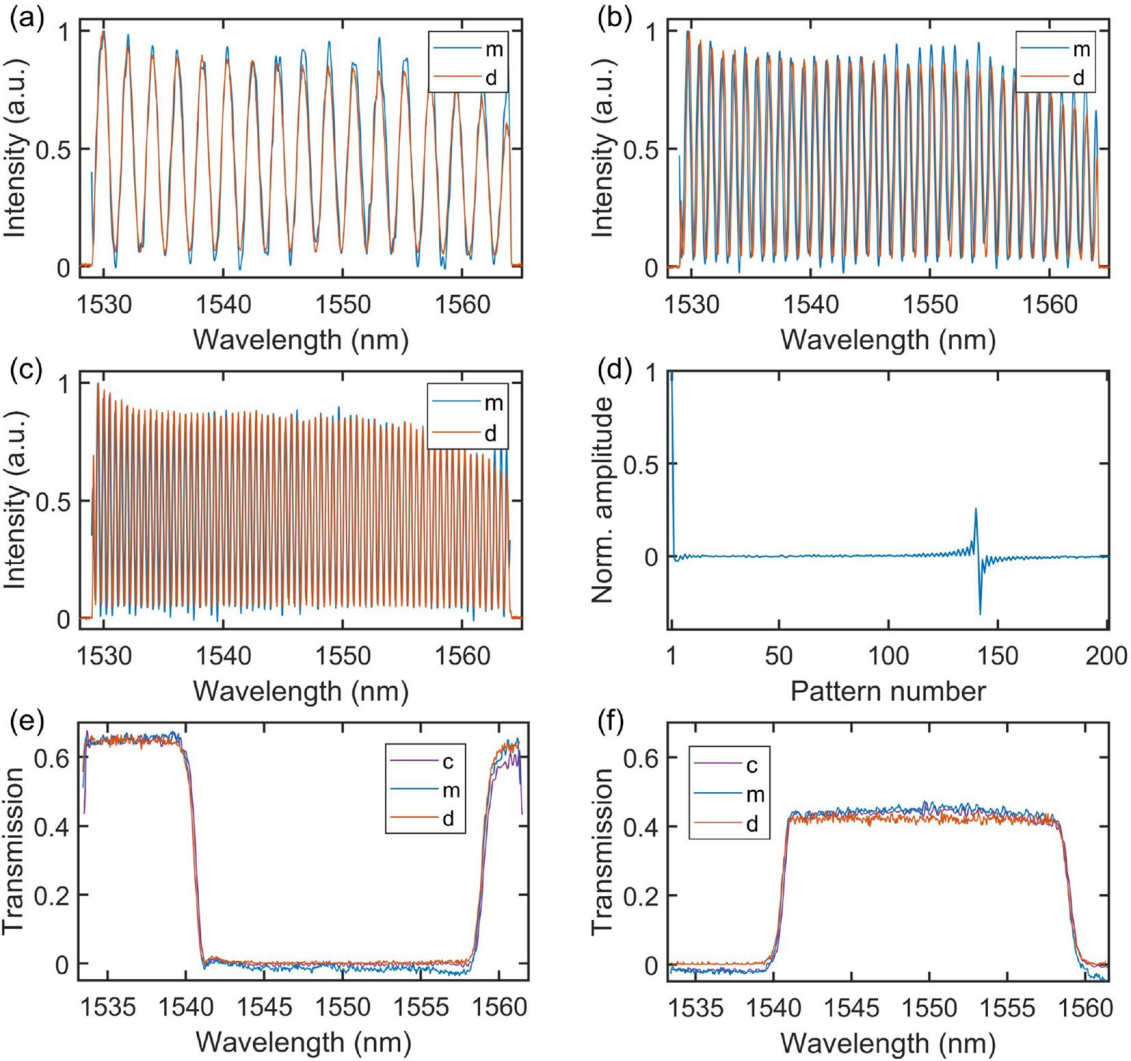
Measured transmission spectra of an unequal path Michelson interferometer with 4 ps (**a**), 8 ps (**b**) and 16 ps (**c**) temporal delay (optical path differences of 1.2 mm, 2.4 mm and 4.8 mm, respectively) corresponding to a spectral modulation period of 2 nm, 1 nm, and 0.5 nm, respectively. (**d**) The signal measured by the integrating single-pixel detector as a function of the modulation pattern frequency for the case of 16 ps delay. Transmission of a wavelength-division multiplexer measured in rejection (**e**) and transmission (**f**) configurations. The solid lines marked as “c”,“m” and “d” correspond to the spectral transmission obtained from computational ghost imaging measurements using ideal complementary patterns, computational ghost imaging measurements using the measured complementary patterns at the waveshaper output, and direct measurement using the OSA, respectively.

The experimentally measured Fourier patterns (with the OSA) are compared against ideal sinusoidal modulations in Fig. 2. Although in the measurements reported below we used a total of 402 patterns (N = 100), here for clarity we only plot the first 61 patterns (including the unmodulated SLED spectrum plus the first 30 sine and cosine modulations). One can see how the period of all the programmed spectral modulations match very well with ideal spectral sinusoidal modulations. We can see that the modulation contrast drops as the modulation frequency (wavelength) increases (decreases), which is caused by the limited operation bandwidth of the programmable filter. This bandwidth limitation constitutes the main limitation for the measurement resolution. In the subsequent proof-of-concept measurements that we report below in Fig. 3, we limit the truncated series to a number of patterns yielding a resolution of 0.3 nm corresponding to the minimum modulation period that can be imposed by the waveshaper without significant drop of contrast. We first probed the spectral response of an unequal path Michelson interferometer known to be a purely sinusoidal function of frequency with modulation period inversely proportional to the temporal delay between the two interferometer arms. In this case, one expects that only the probing sinusoidal patterns (sine and/or cosine) with modulation period corresponding to the temporal delay between the two arms will yield a higher than average intensity signal on the single-pixel detector. In order to verify this, we conducted a series of measurements where we probed the spectral transmission of the Michelson interferometer with complementary Fourier patterns for different optical path differences between the two arms. In this set of measurements, we have used $N=100$ and 36 nm bandwidth (1528–1564 nm), corresponding to an effective spectral resolution of 0.36 nm. The DAQ card operated at 1 MHz sampling rate with 1 s averaging for each modulated pattern. Note that the measurement speed is further limited by the switching time in the programmable filter (500 ms between consecutive patterns) over which data collection is held. The results are shown in Fig. 3 for temporal delay of 4 ps (a), 8 ps (b) and 16 ps (c). The amplitude of the signal as measured by the single-pixel detector, in case of 16 ps delay, for all patterns is also plotted in the figure (see Fig. 3d). One can see that for all the optical path differences, the modulation period and phase are correctly retrieved and that, as expected, one only observes high signal at the single-pixel detector for a modulation frequency corresponding to the inverse of the optical path difference between the two arms of the interferometer. We also note the presence of additional components caused by the non-uniform transmission of the interferometer but these can be also be seen in the reference OSA measurement. We next performed measurements of the spectral transmission/reflection a wavelength-division multiplexer with sharp features. Here, we limited the SLED spectrum to 28 nm bandwidth from 1534 to 1562 nm while keeping $N=100$, corresponding to an effective spectral resolution of 0.28 nm. The results using the pre-measured complementary Fourier patterns are shown in Fig. 3(e,f) along with a reference measurement performed with the OSA for comparison. For completeness, we also show in the figure the results obtained using ideal sine and cosine functions (i.e. not pre-measured) to reconstruct the transmission of the multiplexer output ports. We can see very good agreement between both computational ghost imaging technique approaches (i.e. using ideal mathematical functions or pre-measured patterns) with the OSA reference measurement in terms of amplitude and bandwidth as well as at the band edges where the transmission slope is steepest. This means that in principle one does not even need to pre-measure and store the Fourier patterns in a computer and the reconstruction can be simply performed using theoretical complementary functions. We do note a slight increase in the noise amplitude of the computational measurements as compared to that of the OSA which we attribute to the accumulated error during the spectral response retrieval calculation. The fact that one uses a truncated Fourier series expansion can also artificially smooth the retrieved spectral response and this can be seen when comparing the residual modulation on top of the stop/pass band as measured by the OSA and that retrieved from the computational imaging measurement.

**Figure 4:**
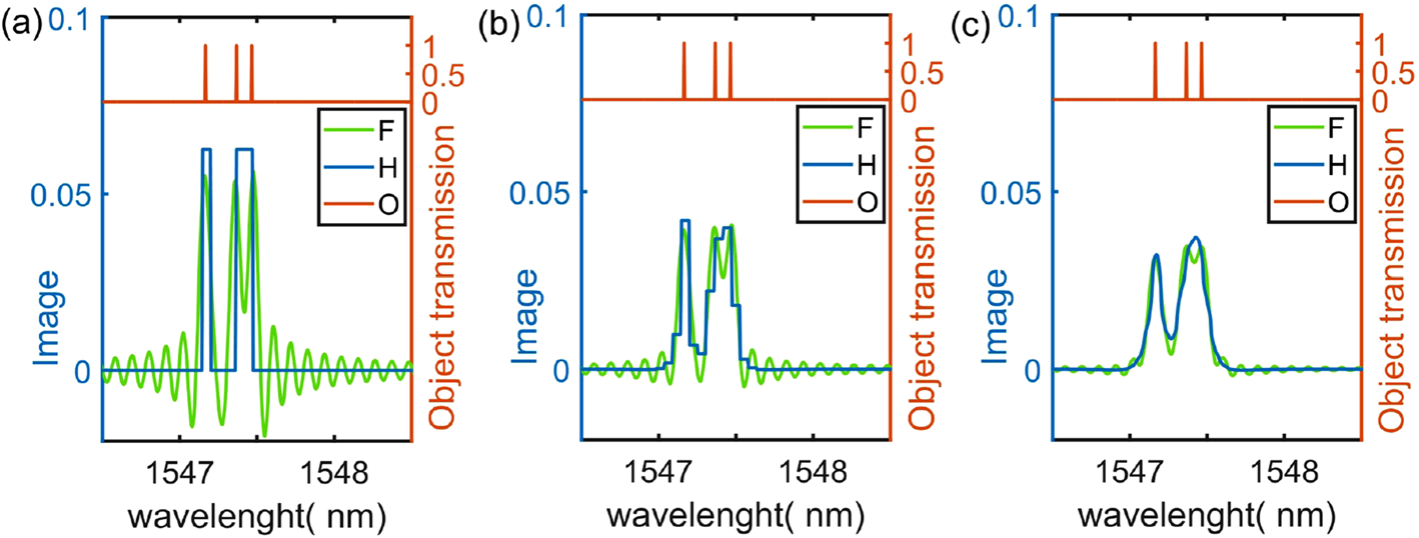
Numerical simulation of computational spectral-domain ghost imaging of an object (O) consisting of three narrow lines of 4 pm width separated by 0.3 nm and 0.1 nm. The retrieved transmission when applying Fourier (F) and Hadamard (H) patterns is shown for different cases: (**a**) mathematically ideal patterns, (**b**) patterns filtered by a modulation frequency limit of the programmable filter (similar to that shown in Fig. 2b applied to the object and mathematically ideal patterns for transmission retrieval, (**c**) patterns filtered by a modulation frequency limit of the programmable filter applied to the object and for transmission retrieval.

In our experiments we have used complementary Fourier patterns, but in principle other types of functions such as e.g. Hadamard patterns could be used. In order to examine whether the particular functional form of the patterns affects the performance of the proposed scheme, we compare numerically in Fig. 4 the retrieved spectral transmission of an artificial object by spectral-domain ghost imaging using Fourier and Hadamard probing patterns. An object with narrow linewidth features and small wavelength separation was specifically chosen in order to compare the resolving power when using a programmable filter with limited modulation bandwidth such as that used in our experimental implementation. The object spectral transmission shown in the top of the figure sub-panels consists of three narrow lines with 4 pm linewidth separated by 0.3 nm and 0.1 nm, respectively. We use a truncated series of 256 applied patterns with the smallest modulation period of 0.1 nm.

Figure 4a shows the retrieved spectral transmission for mathematically ideal applied patterns, i.e. with no additional modulation bandwidth limitation arising from a programmable filter. Figure 4b,c shows the retrieved spectral transmission when bandwidth-limited patterns (corresponding to a modulation wavelength cut-off similar to that of our programmable filter) are applied to the object. (b) corresponds to the case when mathematically ideal patterns are used in the retrieval while (c) uses the (bandwidth-limited) applied patterns also for the retrieval. One can see that in all the illustrated cases, the sharp lines of the object are not fully resolved. This is because the smallest modulation period of the applied patterns is much larger than the actual linewdith of the spectral features. This highlights the fact that the technique can only fully resolved spectral features with linewdith larger than that of the smallest modulation pattern of the applied series, independently of the type of patterns. When mathematically ideal functions are applied, one can also see that Fourier patterns generate artefacts in the form of oscillations in spectral regions where the object does not transmit light, but at the same time they yield better resolution as compared to Hadamard patterns with the two closest spectral lines being distinguished. When the modulation bandwidth is limited by the programmable filter, the resolution decreases and the amplitude of the artefacts induced by the Fourier patterns is also reduced. But even in this case Fourier patterns still allow for distinguishing the narrow line features while Hadamard patterns do not. We performed additional tests (not shown here) including artificial noise added to the simulations and found similar noise figures in terms of average root-mean-square for both Fourier and Hadamard probing patterns.

## Discussion and conclusion

We have demonstrated computational ghost imaging in the spectral domain using complementary Fourier-pair probing patterns. The technique allows for direct measurement of the Fourier coefficients of the object’s spectral response with fast reconstruction. The resolution of the technique is limited by the highest frequency (shortest wavelength) modulation period used in the experiment, corresponding here to a sinusoidal modulation pattern with a period of c.a 0.3 nm beyond which the modulation visibility drops significantly. The technique can be extended to any wavelength region, in particular to the mid-infrared where detectors are not particularly sensitive. There is a wide range of broadband LEDs or supercontinuum sources available in the mid-infrared, and the same type of liquid crystal on silicon technology as used in our filter has also been demonstrated for wavelengths beyond 5 $\upmu$m^25^.

The fact that the modulation is directly applied onto the light source yields several advantages. It allows for uniform spectral illumination across the spatial beam profile, pre-compensation of the light source spectral intensity variations, and removes the need for complex optics to collect and modulate light possibly leading to better signal-to-noise ratio. In experimental schemes where spectral modulation is performed after the object, light may need to be coupled into a fiber or into a spectrometer slit. This is not the case with our approach, resulting in increased intensity collected by the wavelength-integrating detector and enhanced signal-to-noise ratio. This could be particularly beneficial if the object is strongly scattering and the spatial coherence of the beam is significantly degraded, or in the presence of strong aberrations in the collecting optics before the detector. Another advantage of modulating directly the light source rather than light transmitted through the object is that the measurements are not affected by distortion that may occur after the object (e.g. due to nonlinear effects).

In our experiments, the measurement speed is limited by the relatively slow switching speed of the liquid crystals in the programmable filter. As compared to using random probing patterns^7,8^, the speed difference lies essentially in the data collection and processing times. Although the generation of random patterns is significantly faster than that of pre-programmed patterns since no active electrical or optical control is needed, orders of magnitude more realizations are required with random patterns in order to obtain a similar signal-to-noise ratio. Furthermore, unlike random patterns which requires post-processing of the recorded data (see Refs.^7,8^) pre-programmed patterns allows for fast spectral response retrieval by inverse transform. Increasing the measurement speed with pre-programmed patterns could be achieved by dispersing the broadband light and modulating individually the spectral components using a digital mirror array similarly to the arbitrary waveform generator proposed in^26^.

The use of complementary Fourier patterns further reduces the influence of parasitic stray light which can be particularly useful in the case of remote measurements. Although here we use complementary Fourier spectral illumination patterns, one can in principle use other types of patterns such as e.g. Hadamard functions which are traditionally employed in compressed sensing and which can be more suitable for specific applications. The sensitivity of the measurements may also be increased by adding lock-in detection to the current setup and the concept can be extended to 2D hyperspectral measurement, where one acquires different images of a physical object corresponding to different spectral illumination patterns using simply an unfiltered camera. In this case, our approach may be particularly beneficial requiring only a single programmable filter. Our results could open up new perspectives for remote spectral measurements in industrial, biological or security applications, e.g. by illuminating a target with programmed spectral patterns and detecting the reflected signal with an integrating detector without any spectral resolution.
